# Intramuscular Promethazine Added to IV Ondansetron for the Prevention of Intrathecal Morphine-Induced Pruritus and Nausea After Cesarean Delivery: A Randomized Controlled Trial

**DOI:** 10.7759/cureus.99408

**Published:** 2025-12-16

**Authors:** Adam L Wendling, Jonathin N Cates, Cynthia Garvan, Brandon Lopez, M. Anthony Cometa, Matt Andoniadis, Tammy Euliano

**Affiliations:** 1 Department of Anesthesiology, University of Florida College of Medicine, Gainesville, USA; 2 Department of Critical Care, Lexington Medical Center, Lexington, USA; 3 Department of Anesthesiology, BayCare Medical Group, Clearwater, USA

**Keywords:** anesthesia for cesarean delivery, anesthesia spinal, : intrathecal morphine, obstetric anesthesia, pruritus, quality of recovery

## Abstract

Intrathecal morphine is commonly used to facilitate postoperative pain control after cesarean delivery (CD). However, pruritus, nausea, and vomiting are frequent medication-related side effects. We hypothesized that adding prophylactic intramuscular promethazine (IMP) would reduce intrathecal morphine-related pruritus and other side effects more effectively than standard care with ondansetron alone. In this single-center, double-blinded, randomized controlled trial of patients undergoing scheduled CD under spinal anesthesia with intrathecal morphine, all participants received standard care with prophylactic IV ondansetron prior to surgery and were randomly assigned to receive either IMP 25 mg or intramuscular saline placebo after umbilical cord clamping. Pruritus, nausea and vomiting, agitation, fatigue, and pain were assessed at one hour, four hours, and 24 hours postoperatively. Secondary outcomes included pain, the Sedation-Agitation Scale, and fatigue. Sixty-seven patients completed the protocol: 34 received IMP, and 33 received intramuscular saline placebo. Among the total population, 15% reported nausea or vomiting, and 63% reported pruritus. No differences were observed between patients receiving prophylactic IMP and those receiving saline placebo regarding pruritus, nausea, vomiting, agitation, fatigue, or pain at any time point. The addition of prophylactic IMP to standard care with prophylactic IV ondansetron did not reduce the incidence of intrathecal morphine-induced side effects after scheduled CD.

## Introduction

Intrathecal morphine is a common and straightforward method for providing postoperative analgesia after cesarean delivery (CD). However, it frequently induces prolonged pruritus, nausea, and vomiting for up to 48 hours post-administration. Antihistamines such as diphenhydramine have little to no efficacy in treating intrathecal morphine-induced side effects (ITMSEs), may increase sedation, and may theoretically interfere with the initiation of lactation [1-3]. Mu-opioid antagonists such as naloxone effectively treat ITMSE but can reduce analgesic effectiveness. Additionally, due to the short half-life of naloxone, continuous infusions may be necessary to prevent recurrence of symptoms. The related agent nalbuphine, an opioid agonist/antagonist, has been found effective in treating pruritus but may increase sedation and, potentially, nausea [4-7].

Promethazine is a phenothiazine derivative with multiple mechanisms of action. It is a potent peripheral H1 antagonist that directly inhibits the vasodilatory and pruritic effects of histamine. Beyond this, promethazine has central nervous system effects, including antagonism of serotonin, acetylcholine, and histamine receptors, and potential agonism of central alpha-adrenergic receptors, which may contribute to its antiemetic properties. Three studies have evaluated promethazine for the prevention and treatment of ITMSE, with two demonstrating benefit and one showing no effect [8-10]. These studies did not evaluate combinations of medications with different mechanisms of action. Combination therapy for preventing postoperative nausea and vomiting is recommended as part of enhanced recovery protocols after CD [11]. Given promethazine’s multiple central mechanisms beyond H1 antagonism, it is plausible that adding promethazine to a 5-HT3 antagonist could further reduce ITMSE.

We hypothesized that intramuscular promethazine (IMP) added to standard care would reduce the severity of ITMSE compared with a serotonin antagonist alone. To test this, we conducted a double-blind, placebo-controlled, randomized trial to evaluate whether prophylactic IMP in addition to standard care with IV ondansetron could reduce ITMSE in patients undergoing elective CD under spinal anesthesia with intrathecal morphine. Our primary objective was to determine whether the addition of IMP to ondansetron reduces pruritus immediately after CD. Secondary outcomes included pruritus severity at four and 24 hours postoperatively, as well as incidence of nausea and vomiting, sedation, and pain at one, four, and 24 hours after study drug administration between patients receiving IMP plus IV ondansetron versus placebo plus IV ondansetron.

## Materials and methods

After institutional review board approval (IRB202002297), patients scheduled for CD were contacted more than one day prior to surgery for recruitment into this study. The study was registered with ClinicalTrials.gov (NCT04805073), and the complete data set is available in the Mendeley Data repository [12]. Inclusion criteria were age 18 years or older and planned CD under spinal anesthesia. Exclusion criteria included incarceration, inability to communicate with investigators, allergy or contraindication to morphine or promethazine, contraindication to spinal anesthesia, and prolonged QTc (>500 ms). Informed consent was obtained from all participants prior to surgery.

All patients received 975 mg of acetaminophen orally and a nonparticulate antacid before arrival in the operating room. All patients received ondansetron 4 mg IV prior to spinal anesthesia. Spinal anesthesia consisted of hyperbaric bupivacaine 12 mg, fentanyl 20 mcg, and preservative-free morphine 100 mcg, administered via an atraumatic 25-gauge spinal needle. A prophylactic phenylephrine infusion, along with intermittent boluses of phenylephrine, ephedrine, and/or glycopyrrolate, was used to maintain blood pressure within 20% of baseline. IV fluid administration was at the discretion of the anesthesia team. The remainder of the surgical procedure, including prophylactic antibiotics, surgical technique, and uterotonic medications, followed institutional standard.

During surgical closure, one of the investigators administered an intramuscular injection of 1 mL containing either 0.9% saline placebo or promethazine 25 mg into the patient’s left thigh. Randomization and study medication preparation were provided by an independent investigational pharmacy not otherwise involved in the study. Study medication was supplied in identical 1 mL syringes. All study personnel and patients were blinded to treatment.

Postoperatively, all patients received scheduled IV ketorolac for 24 hours, followed by oral ibuprofen 600 mg every six hours and oral acetaminophen 650 mg every six hours. For breakthrough pain greater than 4 on the Defense and Veterans Pain Rating Scale (DVPRS) [13], patients received oxycodone 5 mg orally every four hours as needed. Rescue antiemetics were administered in the following order if needed: ondansetron 4 mg IV, metoclopramide 5 mg IV, or promethazine 6.25 mg IV. For pruritus, patients could receive rescue nalbuphine 2.5 mg IV every six hours as needed.

All patients rated the severity of pruritus on an 11-point Likert scale (0 = no itching, 10 = worst imaginable pruritus) at one, four, and 24 hours after study drug administration [14]. Patients also rated nausea and vomiting (yes/no), agitation/fatigue (Richmond Agitation-Sedation Scale (RASS)), and pain (DVPRS) at the same time points [15]. The DVPRS and RASS scales are freely available for use by clinicians and researchers [16,17].

The original planned sample size was 50 patients per treatment arm, providing >80% power to detect a 20% or greater difference on the pruritus scale at a two-sided 0.05 significance level [18]. During the study, a new US FDA warning was issued for IV promethazine, leading our hospital system to remove all parenteral promethazine from clinical care areas. We recalculated power based on patients who had already enrolled or would complete enrollment before the removal of parenteral promethazine. With a total of 42 patients (21 per group), the study had an 81% probability of detecting a treatment difference at a two-sided 0.05 significance level, assuming a true difference of 2.4 units on the pruritus scale (equivalent to the difference between none and moderate itching or mild and severe), with an SD of 2.645. At the time the study was stopped, an additional 25 patients were enrolled. Sample size calculations were based on a calculator developed by David A. Schoenfeld, PhD, Massachusetts General Hospital, Mallinckrodt General Clinical Research Center, National Institutes of Health, National Center for Advancing Translational Sciences.

Ordinal measures of itching and agitation/sedation were compared between groups using Wilcoxon’s rank-sum test. Presence or absence of nausea/vomiting was compared using Fisher’s exact test. For reporting, pruritus scores were dichotomized into (1) none (score = 0), (2) mild (scores 1-2), (3) moderate (scores 3-4), and (4) severe (scores ≥5). All tests were two-sided with significance set at 0.05. SAS version 9.4 (Cary, NC, USA) was used for statistical analyses, and R version 4.4.1 (Vienna, Austria) was used for graphing.

## Results

**Figure 1:**
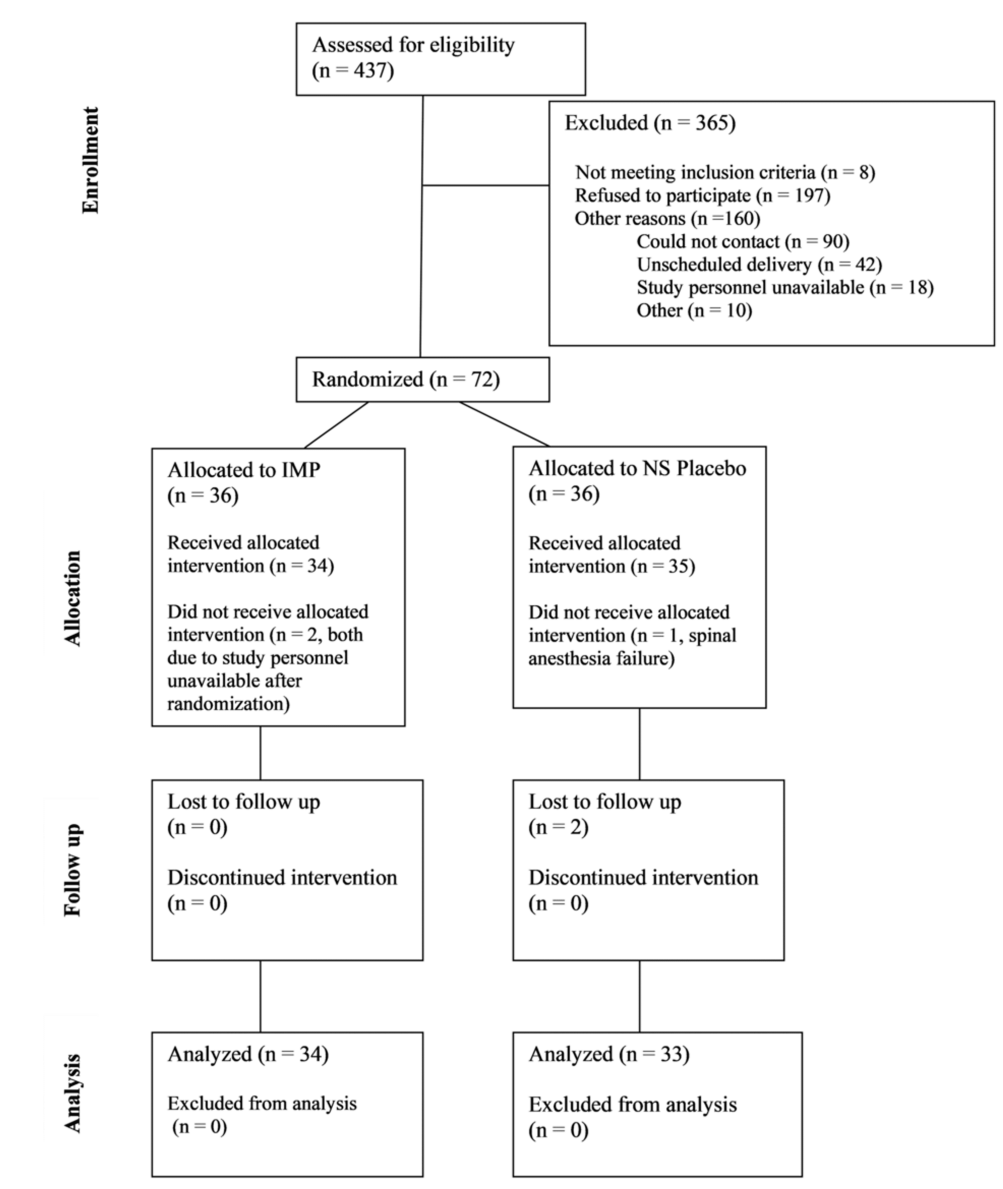
CONSORT diagram Source: [19]

A total of 67 women completed the study (34 in the promethazine group and 33 in the saline group), with a mean (SD) age of 31.6 (5.0) years (Figure 1) [19]. Data were missing for some participants at specific time points: two women at one hour only, four women at four hours only, two women at 24 hours only, and one woman at both four and 24 hours.

Among the total study population, 63% (95% CI: 50%-75%) reported some degree of itching, and 15% (95% CI: 8%-26%) reported symptoms of nausea and/or vomiting. Immediately postpartum, 37% (n = 24) of patients reported no itching, 25% (n = 16) had mild itching, 20% (n = 13) had moderate itching, and 18% (n = 12) reported severe itching. Immediate postpartum measures of nausea/vomiting, itching, and agitation/sedation are summarized in Table 1. No significant differences were observed between the promethazine and saline groups at one hour after study drug administration. 

**Table 1:** One-hour overall and group comparisons of nausea/vomiting, itching, and RASS Itching was assessed using an 11-point Likert scale [14]. Agitation and sedation were assessed using the RASS [15]. Presence or absence of nausea/vomiting was compared using Fisher’s exact test. Itching and agitation/sedation scores were compared using Wilcoxon’s rank-sum test. Data were missing for two patients in the saline placebo cohort. IMP, intramuscular promethazine; RASS, Richmond Agitation-Sedation Scale

Measure	Overall, (%) n	Saline, (%) n	IMP, (%) n	p-Value
Nausea				0.5
No	85% (55)	81% (25)	88% (30)	
Yes	15% (10)	19% (6)	12% (4)	
Itching				0.876
None	37% (24)	35% (11)	38% (13)	
Mild	25% (16)	23% (7)	26% (9)	
Moderate	20% (13)	19% (6)	21% (7)	
Severe	18% (12)	23% (7)	15% (5)	
Agitation and sedation				0.565
Alert and calm	75% (49)	77% (24)	74% (25)	
Drowsy	23% (1)	19% (6)	26% (9)	
Restless	2% (1)	3% (1)	0% (0)	

At four hours postpartum, 0% of patients in the promethazine group reported nausea/vomiting compared with 16% in the saline group (p = 0.053). No significant differences were observed between groups in measures of itching or agitation/sedation (Table 2).

**Table 2:** Four-hour overall and group comparisons of nausea/vomiting, itching, and RASS Itching was assessed using an 11-point Likert scale [14]. Agitation and sedation were assessed using the RASS [15]. Presence or absence of nausea/vomiting was compared using Fisher’s exact test. Itching and agitation/sedation scores were compared using Wilcoxon’s rank-sum test. Data were missing for five patients at this time point (two from the saline placebo cohort and three from the IMP cohort). IMP, intramuscular promethazine; RASS, Richmond Agitation-Sedation Scale

Measure	Overall, (%) n	Saline, (%) n	IMP, (%) n	p-Value
Nausea				0.053
No	92% (57)	84% (26)	100% (31)	
Yes	8% (5)	16% (5)	0% (0)	
Itching				0.566
None	34% (21)	29% (9)	39% (12)	
Mild	27% (17)	29% (9)	26% (8)	
Moderate	16% (10)	13% (4)	19% (6)	
Severe	23% (14)	29% (9)	16% (5)	
Agitation and sedation				0.255
Alert and calm	87% (54)	94% (29)	81% (25)	
Drowsy	13% (8)	6% (2)	19% (6)	
Restless	0% (0)	0% (0)	0% (0)	

**Figure 2:**
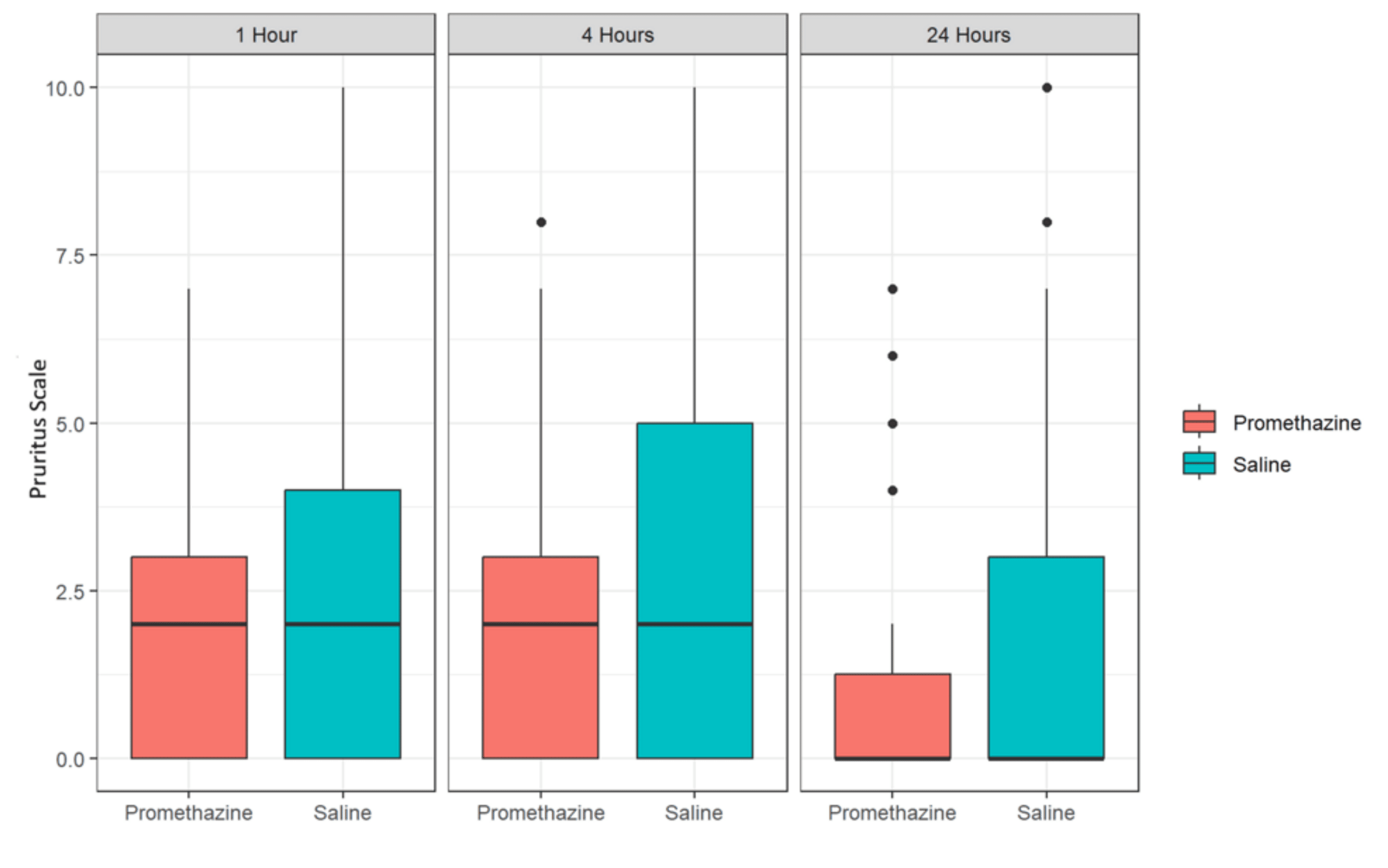
Box plot depicting mean and SD of pruritus scores at one, four, and 24 hours after study drug administration Source: [14]

At 24 hours postpartum, no differences were observed between groups in measures of itching, nausea/vomiting, or agitation/sedation (Table 3). Nausea/vomiting rates decreased over time: 15% (n = 10) at one hour, 8% (n = 5) at four hours, and 2% (n = 1) at 24 hours. Similarly, pruritus decreased over time, as shown in Figure 2. Alertness rates increased over time: 75% (n = 49) at one hour, 87% (n = 54) at four hours, and 98% (n = 63) at 24 hours.

**Table 3:** 24-hour overall and group comparisons of nausea/vomiting, itching, and RASS Itching was assessed using an 11-point Likert scale [14]. Agitation and sedation were assessed using the RASS [15]. Presence or absence of nausea/vomiting was compared using Fisher’s exact test. Itching and agitation/sedation scores were compared using Wilcoxon’s rank-sum test. Data were missing for three patients at this time point (one from the saline placebo cohort and two from the IMP cohort). IMP, intramuscular promethazine; RASS, Richmond Agitation-Sedation Scale

Measure	Overall, (%) n	Saline, (%) n	IMP, (%) n	p-Value
Nausea				1
No	98% (63)	97% (31)	100% (32)	
Yes	2% (1)	3% (1)	0% (0)	
Itching				0.371
None	61% (39)	53% (17)	69% (22)	
Mild	14% (9)	16% (5)	13% (4)	
Moderate	9% (6)	16% (5)	3% (1)	
Severe	16% (10)	16% (5)	16% (5)	
Agitation and sedation				1
Alert and calm	98% (63)	100% (32)	97% (31)	
Drowsy	2% (1)	0% (0)	3% (1)	
Restless	0% (0)	0% (0)	0% (0)	

## Discussion

Intrathecal morphine provides prolonged analgesia after CD with minimal systemic drug exposure and is recommended as part of enhanced recovery protocols after CD [11]. However, ITMSEs are common. In our study, 15% of patients experienced nausea and/or vomiting, and 63% experienced pruritus. The mechanism by which neuraxial morphine induces these side effects remains unclear. Multiple medications have been tested through various routes to prevent and treat ITMSE [20]. Safe and easily administered methods that do not compromise recovery by causing sedation, dysphoria, or reducing analgesia are particularly important. Opioid agonist-antagonists may cause some degree of sedation, dysphoria, and reduced analgesia from intrathecal morphine [4-7]. Other medications, sometimes administered via routes not approved in the United States, have shown some utility in reducing ITMSE [20].

IV ondansetron is often administered primarily for its antiemetic properties but has also been reported to reduce pruritus [21-23]. Two of three previous studies demonstrated that promethazine can prevent or treat ITMSE. Slappendel et al. administered IMP to treat pruritus after varying doses of intrathecal morphine in orthopedic patients [8]; only one of 143 patients required escalation of therapy beyond IMP. Eldor et al. conducted a randomized controlled trial evaluating prophylactic IMP for preventing pruritus in patients undergoing CD with epidural morphine [9]. Forty patients were randomized to receive either placebo or IMP 25 mg at the time of administration of 3.5 mg of epidural morphine at the conclusion of CD. None of the patients receiving IMP experienced pruritus, whereas 35% of the control group required rescue treatment. Horta examined the prophylactic potential of four drugs, including promethazine, in 300 women undergoing elective CD [10]. In this study, IV promethazine did not prevent ITMSE. Vice-O’Con et al. reviewed medications effective for ITMSE treatment in 2018 and reported that IV promethazine did not provide relief but did not address intramuscular administration or combination therapies [24]. Singh et al. performed a systematic review and network meta-analysis of pharmacologic agents to prevent pruritus after neuraxial morphine during CD [20], identifying propofol, neuraxial and systemic opioid agonist/antagonists, opioid antagonists, neuraxial dopamine antagonists, and serotonin antagonists as more effective than placebo. Concerns remain regarding the neuraxial administration of certain medications, such as dopamine antagonists, which are not approved for this route in the United States.

Notably, the two studies that demonstrated benefit with promethazine administered the medication intramuscularly [8,9]. Intramuscular administration is the safest parenteral route and aligns with current FDA guidance [25,26]. Combination antiemetic therapy is recommended for enhanced recovery. Unlike prior studies, our standard of care included prophylactic IV ondansetron, which has both antiemetic and antipruritic properties. We evaluated whether adding IMP to prophylactic ondansetron could further reduce ITMSE. Our study compared two prophylactic agents (IMP plus ondansetron) versus one (ondansetron alone). No significant differences were observed between groups in pruritus severity (primary outcome), nausea/vomiting, sedation, or pain at one, four, or 24 hours postoperatively. Adding prophylactic IMP 25 mg to ondansetron 4 mg did not provide a clinically meaningful reduction in intrathecal morphine side effects. Patients who received IMP did not experience increased sedation, agitation, or pain compared with ondansetron alone.

This study has several limitations. It is a small, single-center trial. During the study period, a new FDA warning regarding promethazine administration restricted access to the drug in our hospital, although our intramuscular route remained consistent with labeling [26]. We ended the study prior to reaching our originally planned enrollment due to slow recruitment and limited medication availability. With the number of participants who completed the protocol, the study was powered to detect only large differences in itching (e.g., a reduction from severe to moderate or from moderate to none). The RASS scale detects large changes in consciousness and may not capture subtle changes such as mild sedation. Over two-thirds of patients screened declined participation. Common reasons included (1) prior lack of ITMSE with previous CDs; (2) concern about participating in a study; and (3) concern regarding potential sedation. Overall, most patients did not perceive the potential benefit of adding IMP to standard care with ondansetron as worthwhile. Considering these enrollment challenges, drug availability, and prior evidence, a larger study does not appear warranted.

## Conclusions

ITMSEs, including pruritus, nausea, and vomiting, complicate recovery after elective CD and remain common despite prophylactic medications. In this randomized, double-blind, placebo-controlled study, the addition of prophylactic IMP to standard care with prophylactic ondansetron did not significantly reduce the incidence of nausea, vomiting, or pruritus after spinal anesthesia with intrathecal morphine compared with ondansetron alone. The addition of IMP also did not increase sedation or pain.

Use of two prophylactic agents (ondansetron and promethazine) was not superior to a single prophylactic agent (ondansetron alone) in reducing pruritus, nausea, or vomiting after spinal anesthesia with intrathecal morphine for elective CD. Given the lack of additional benefit, combined with regulatory and institutional restrictions on parenteral promethazine and high patient refusal rates, routine prophylactic use of IMP is not recommended in modern enhanced recovery pathways after CD.
